# Forthcoming papers

**DOI:** 10.1155/S1463924686000226

**Published:** 1986


wird die Metabolitenkonzentration in den Perchlorsäure-Extrakten unter Verwendung der Mittelwerte aller oder verschiedener Standardgruppen bestimmt. Im Zusammenfassungsteil werden alle Probeninformationen inklusive Verdünnungsfaktoren, Patientenidentifikation und Kontrollen zusammengefasst und die Resultate in einem kohörenten Format ausgedruckt. Die Korrelation zwischen den Rechnerresultaten und solchen, die in konventioneller Art von Schreiberkurven abgelesen werden, ist ausgezeichnet. Der Zeitaufwand für die Verarbeitung der Daten mit dem Rechner ist wesentlich geringer.

### Page 70

### Automation d'un système à flux d'injection pour multiples échantillons

J. Ruz *et al.*

Un système automatique 'flow injection' qui se base sur l'intégration de deux détecteurs (photométriques et potiontiométriques) est présenté. Ce système permet de collecter des données d'absorption et de pH de chaque échantillon moyennant un microprocesseur en ligne, qui, avec le programme de calcul nécessaire, procure la concentration de 9 espèces différentes de chrome présentes dans l'échantillon. Une configuration 'renversé' et un mode de mesure utilisant les zones asymétriques permet la discrimination entre les différents état d'oxydation du chrome.

### Automatisierung eines FIA-Systems für die Mulitkomponenten-analyse

J. Ruz *et al.*

Ein automatisiertes Fliessinjektionsysstem, in welchem zwei Detektoren (photometrisch und potentiometrisch) integriert sind, erlaubt gleichzeitig mit einem Mikroprozessorsystem pH- und Absorptionsdaten zu erfassen. Mit einem geeigneten Rechenprogramm können damit die Konzentrationen von bis zu neun Arten von Chrom in der Probe bestimmt werden. Eine reversierte Konfiguration und das asymmetrische Mündungszonen-Vehalten werden für die Unterscheidung der verschiedenen Oxydationszustände des Chrom benützt.

---

### FORTHCOMING PAPERS

*Later issues of the Journal will contain the following articles:*

Time utilities: programs in MUMPS for calculating intervals of time and for timing events
*T. G. Pellar and A. R. Henderson*

A model for cost analysis—application to clinical laboratory test economics using computer facilities
*F. R. Hindriks, A. Bosman, Chr. Frowein, H. Kamps and W. van der Slik*

Biochemical laboratory management with a microcomputer
*R. Kowalezyk, G. Morgant, F. Ch. Baumann and J. Giboudeau*

Evaluation of a new semi-automated high-performance liquid chromatography method for glycolysated haemoglobins
*A. Carpinelli, A. Mosca and P. Bonini*

An evaluation of the Waters Pico-Tag system for the amino-acid analysis of food materials
*J. A. White, R. J. Hart and J. C. Fry*

Serum ionized calcium: evaluation of the analyte +2 calcium analyser in a clinical chemistry laboratory
*R. Freaney, O. M. Casey, T. Quinn and F. P. Muldowney*

Kinetic-based determinations in continuous-flow analysis
*M. D. Luque de Castro and M. Valcarcel*

Comparative evaluation of a Technicon SMAC2/RA1000 System with an American Monitor Parallel during normal service work
*A. J. Little et al.*

---

### NOTES FOR AUTHORS

Two copies of articles should be submitted to the Editor. All articles should be typed in double spacing with ample margins, on one side of the paper only. The following items should be sent: (1) a title-page including a brief and informative title, avoiding the word 'new' and its synonyms; a full list of authors with their affiliations and full addresses; (2) an abstract of about 250 words – this should succinctly describe the scope of the contribution and highlight significant findings or innovations; (3) the main text; (4) appendices (if any); (5) references; (6) tables, each table on a separate sheet and accompanied by a caption; (7) illustrations (diagrams, drawings and photographs) numbered in a single sequence from 1 upwards and with the author's name on the back of every illustration; captions to illustrations should be typed on a separate sheet.

### References

References should be indicated in the text by numbers following the author's name, i.e. Skeggs [6].

### Illustrations

Original copies of diagrams and drawings should be supplied, and should be drawn to be suitable for reduction to the page or column width of the *Journal*, i.e. to 85 mm or 179 mm, with special attention to lettering size.

### Proofs and offprints

The principal or corresponding author will be sent galley proofs for checking and will receive 50 offprints free of charge.

*Manuscripts should be sent to either Dr P. B. Stockwell or Ms M. R. Stewart, see cover.*

